# Improving healthcare accessibility for pregnant women and children in the context of health system strengthening initiatives and terrorist attacks in Central Mali: a controlled interrupted time series analysis

**DOI:** 10.1136/bmjgh-2023-012816

**Published:** 2024-05-02

**Authors:** David Zombré, Dansiné Diarra, Laurence Touré, Emmanuel Bonnet, Valery Ridde

**Affiliations:** 1 Evaluation and Data Analytics, Recherche pour la santé et le développement 04 BP 8398 Ouagadougou 04, Arrondissement 6, Secteur 28, Ouagadougou, Burkina Faso; 2 Faculté d'Histoire et de Géographie, Université des Sciences Sociales et de Gestion de Bamako, Bamako, Mali; 3 Association Malienne de Recherche et Formation en Anthropologie des dynamiques locales, MISELI, BP E5448, Bamako, Mali; 4 Résiliences, Institut de recherche pour le developpement, bondy, Seine Saint Denis, France; 5 CEPED, IRD, Paris, France; 6 ISED, UCAD, Dakar, Senegal

**Keywords:** Maternal health, Child health, Health services research

## Abstract

**Introduction:**

The Health and Social Development Program of the Mopti Region (PADSS2) project, launched in Mali’s Mopti region, targeted Universal Health Coverage (UHC). The project addressed demand-side barriers by offering an additional subsidy to household contributions, complementing existing State support (component 1). Component 2 focused on supply-side improvements, enhancing quality and coverage. Component 3 strengthened central and decentralised capacity for planning, supervision and UHC reflection, integrating gender mainstreaming. The study assessed the impact of the project on maternal and child healthcare use and explored how rising terrorist activities might affect these health outcomes.

**Methods:**

The impact of the intervention on assisted births, prenatal care and curative consultations for children under 5 was analysed from January 2016 to December 2021. This was done using an interrupted time series analysis, incorporating a comparison group and spline regression.

**Results:**

C1 increased assisted deliveries by 0.39% (95% CI 0.20 to 0.58] and C2 by 1.52% (95% CI 1.36 to 1.68). C1-enhanced first and fourth antenatal visits by 1.37% (95% CI 1.28 to 1.47) and 2.07% (95% CI 1.86 to 2.28), respectively, while C2 decreased them by 0.53% and 1.16% (95% CI −1.34 to −0.99). For child visits under 5, C1 and C2 showed increases of 0.32% (95% CI 0.20 to 0.43) and 1.36% (95% CI 1.27 to 1.46), respectively. In areas with terrorist attacks, child visits decreased significantly by 24.69% to 39.86% compared with unexposed areas.

**Conclusion:**

The intervention had a limited impact on maternal and child health, falling short of expectations for a health system initiative. Understanding the varied effects of terrorism on healthcare is key to devising strategies that protect the most vulnerable in the system.

**WHAT IS ALREADY KNOWN ON THIS TOPIC?**

- Strengthening both healthcare supply and demand is crucial for substantial access improvements.
- Terrorist attacks directly impact maternal and child health indicators, causing immediate declines.
- Recurrent terrorist attacks exacerbate insecurity and hinder the effectiveness of interventions designed to strengthen health systems.

**WHAT THIS STUDY ADDS?**

- The intervention targeting maternal and child health outcomes in Mali yielded limited but measurable gains.
- Terrorist attacks significantly attenuated the intervention’s effectiveness on healthcare utilisation.
- The intervention’s limited impact was also related to design flaws, implementation barriers and challenges arising from ongoing terrorist activities.

**HOW THIS STUDY MIGHT AFFECT RESEARCH, PRACTICE OR POLICY?**

- The intervention’s modest impact suggests the need for more effective strategies in conflict-affected environments.
- Collaboration between donors, policymakers and implementers is key to applying implementation science effectively in conflict-affected settings.
- The study emphasises the importance of considering the influence of security contexts in evaluating healthcare interventions.

## Introduction

In the mid-2010s, a French minister decided to accompany the Sahel countries in their journey to universal health coverage.^1^ To this end, in 2013, he launched the Sahel Health Solidarity Initiative (I3S), with 30 million EUR 30, financed by 10% of the revenue from taxation on financial transactions.^2 3^ The aim was to support countries in addressing financial access to healthcare for children and pregnant women. For several decades, significant efforts have been made to strengthen the provision of care in the countries of the Sahel region, particularly in Mali,^4^ without sufficiently acting on the modalities of out-of-pocket payments for health. All the research showed the need to continue the efforts by countries to reduce out-of-pocket costs for patients when they use health facilities.^5^ The aim of this initiative was to support countries committed to policies that address the financial barriers to healthcare.

Despite the international consensus supporting these policies,^6^ Mali, a pioneer in the spread of out-of-pocket payments for health prompted by WHO and UNICEF in the mid-1980s,^7 8^ continues to face challenges in fully implementing them.

While pilot projects have demonstrated the potential of fee exemptions to improve healthcare access,^9 10^ policy response has been limited, resulting in partially implemented exemption schemes. The effectiveness of these partial exemptions remains debatable, with documented evidence suggesting limited impact,^11^ despite specific instances of positive outcomes, such as increased caesarean section uptake.^12 13^

Mali is currently reflecting on its health insurance system and is looking for a solution to cover both the indigent and the informal sectors. For the former, it organised an ambitious policy of free membership in a unremarkable insurance system financed by the State and local authorities and organised by the National Agency for Medical Assistance.^14^ For the latter, it intends to develop its system of community-based health insurance (CBHI). Given the state of international knowledge on the inefficiency of these CBHI, and the challenges of the ability to pay membership by populations,^15 16^ the State decided, as in Senegal,^17^ to subsidise membership at 50%.

While the 1980s and 1090s saw efforts to improve healthcare in Mali, significant gaps remain, demanding further investment.^4 18^ Amidst this underperforming system, the I3S project emerged with partial fee exemptions and 50% CBHI subsidies to boost demand and address affordability. The Health and Social Development Program of the Mopti Region (PADSS2) agreement was established in February 2015, following preidentification and feasibility missions conducted in October 2013 and May–June 2014, respectively. I3S made a 5-million-euro donation to strengthen the demand and accessibility of healthcare. Additionally, an additional 8 million euros from Muskoka funds were allocated to enhance service quality.^2^

Adding further complexity, the I3S implementation coincided with rising terrorist activity in Mali. This phenomenon’s multifaceted impact on maternal and child health service utilisation in low and middle-income countries is well documented.^19–21^ Terrorist attacks not only disrupt healthcare infrastructure and access but also foster instability and fear, discouraging service utilisation. Research in Burkina Faso, for instance, demonstrates a decline in assisted deliveries and caesarean sections due to terrorist activities, highlighting their detrimental effect on healthcare delivery. Moreover, these conditions can worsen pre-existing health issues, such as malnutrition and use of health services among vulnerable populations, thereby placing additional strain on healthcare systems. An illustrative case study from Pakistan demonstrates a correlation between decreased healthcare involvement and elevated rates of child stunting and underweight conditions.^21^

There have been limited studies conducted in the Sahel region that have investigated the collective effect of interventions targeting both the demand and supply aspects, particularly in difficult circumstances.^22 23^

This study seeks to evaluate the impacts of this project on both the supply and demand aspects of maternal and child healthcare in the Mopti region of Mali, while considering the influence of the security context.^24^

This study is notable for its distinctive methodology, as it assesses an intervention that impacts both the demand for and supply of healthcare services simultaneously. Significantly, this intervention takes place in a situation of increased insecurity and rising terrorist attacks, which is a crucial aspect that is seldom examined in current literature.^19^ We recognise the intricate methodological challenges involved in incorporating terrorism into the evaluation of public policies in the Sahel region, a facet that has been given little consideration in previous research. In essence, our research aims to thoroughly analyse these challenges in order to gain a deep understanding of the intricate dynamics at play.

## Methods

### Study setting

The intervention occurred in the Mopti region, which is bordered to the north by the Timbuktu region, to the west and south by the Ségou region, and to the southeast by Burkina Faso. The area of the region spans 79 017 square kilometres within the national territory of Mali. The population, as per the most recent census conducted in 2009, is estimated to be 2 036 209 residents. Agriculture in the Mopti region is flourishing due to the abundant irrigation provided by the Niger River and the Bani River. The fishing industry is a crucial sector. Mopti serves as a pivotal hub for trade and commerce, connecting the northern and southern regions of Mali as well as neighbouring countries. The tourism industry is highly developed in this area, particularly in Djenné, Mopti and the Dogon region.

The field of health administration is categorised into three hierarchical levels: cercle, regional and national. The health circle, also known as the district level, is the operational entity tasked with the planning, allocation of funds, execution and oversight of health initiatives at the primary level. The regional level provides technical support. It oversees the execution of the primary-level programmes. The national-level pertains to the strategic level, where strategic directions are established and decisions regarding investments and operations are made.

The health pyramid is segmented into three levels at the care delivery structures. The operational level consists of two distinct steps. Initially, there exists a network of community health centres (CSComs) overseen by community health associations (ASACO) consisting of individuals who represent the local population.^25^ CSComs serve as the front-line services, providing a minimum package of essential care. The Ministry of Health constructs the facilities, assembles the teams and procures the initial inventories of medications. The individual has the authority to allocate and compensate employees, specifically the technical director, who holds a doctorate degree.^25^ A portion of CSCom expenditures is subsidised by the government and various non-governmental organisations (NGOs), while a substantial amount is covered through direct payment from patients. According to World Health (2014), households in Mali contribute 62% of the overall health expenses. Additionally, the 65 operational district hospitals, which constitute the second tier of the healthcare hierarchy, offer medical services for cases referred from the primary level. These services encompass obstetric emergencies, paediatric emergencies and fundamental surgical procedures. The intermediate level consists of eight Public Hospital Establishments. The central level consists of five University Hospital Centres, with three being general and two being specialised.

### Details of the intervention

The PADSS II was designed to enhance maternal, neonatal and child health as well as family planning services across 57 CSComs in the Mopti and Bandiagara districts, in addition to the regional hospital. These districts were selected due to their significant healthcare needs, which were among the highest in the region. The initiative aimed to address both the supply and demand sides of healthcare through three primary components: improving financial access to healthcare services, enhancing the quality and availability of healthcare provision and building the capacity of healthcare providers and institutions.

The project started in December 2016 and consisted of the rollout of two out of three components: *the first component (C1)* targeted the demand for health services to reduce cultural and financial barriers to healthcare. It accompanied the implementation of the national strategy for extending sickness risk coverage through the deployment of CBHI. It offered an additional subsidy to household contributions (30% in addition to the 50% state subsidy). *The second component (C2)* focused on the provision of care to improve the quality of care and health coverage. *The first part* targeted some CSComs from the Mopti and Bandiagara cercles, which received a support package: support to the planning process, upgrades (infrastructure, equipment, medicines), accreditation mechanism (which was planned but could not be carried out) and support to ASACO on planning. *The second component* involved the drafting and implementation of an Establishment Project for the new district hospital as well as the construction, equipment and establishment of a blood bank service and a sickle cell disease control service. *The third component (C3)* was aimed at strengthening central and deconcentrated capacity in planning and supervision, supporting reflection on Universal Health Coverage, gender mainstreaming in the provision of care and capitalising on innovative experiences tested under the programme.

### Intervention implementation

**Figure 1:**
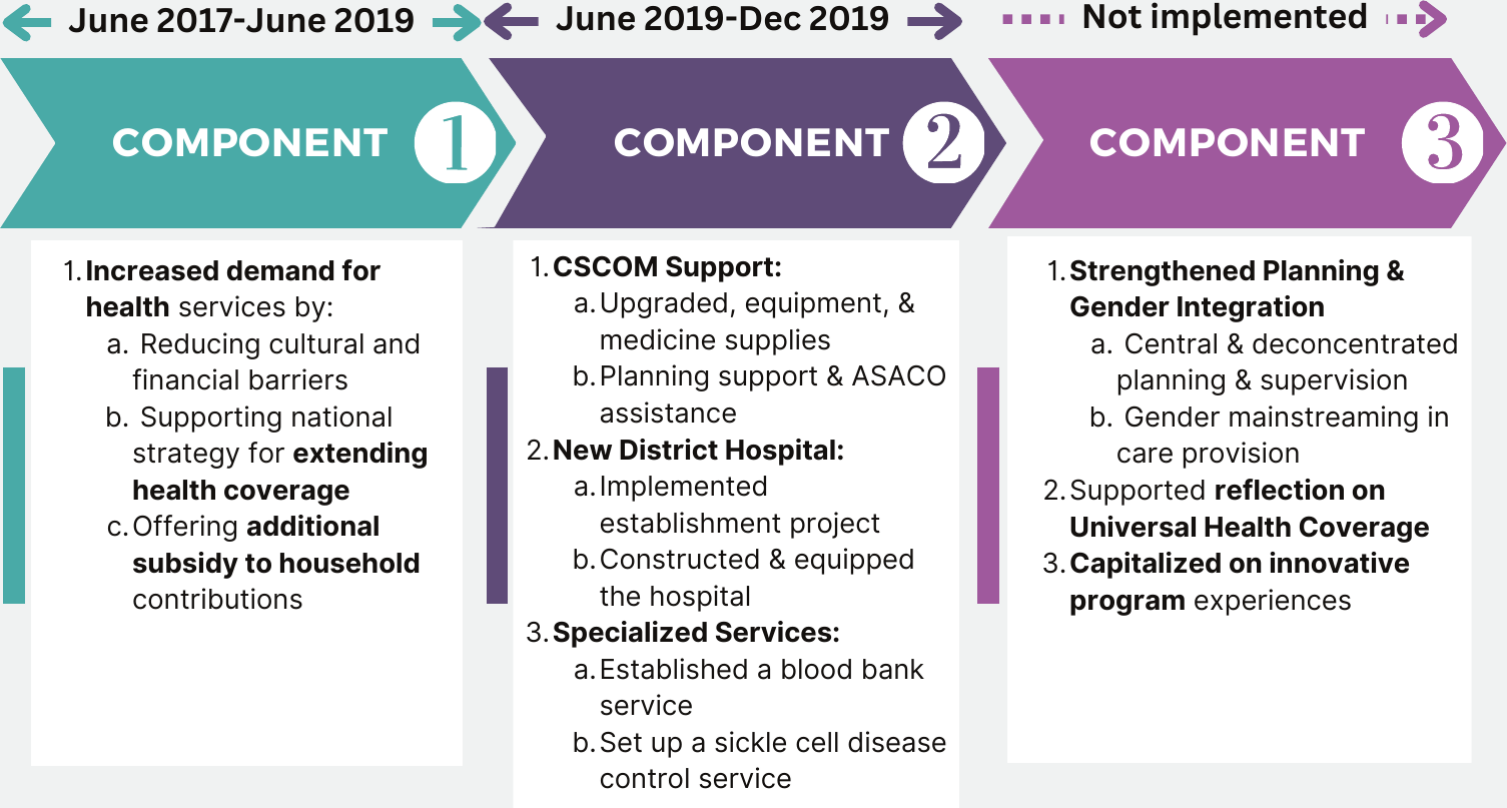
Description of the intervention and its implementation. ASACO, community health associations; CSCOM, community health centres.

The intervention was deployed sequentially and gradually in two phases in 30 CSCom of Bandiagara and 27 CSCom of Mopti (figure 1). There was a variation in the intensity and degree of the implementation of the components between the CSComs. The first phase concerned the implementation of C1 through the deployment of CBHI and the subsidisation of premiums. It started in 16 CSCom in June 2017 and ended in June 2019. The second phase focused on the C2 implementation through the rehabilitation of 21 CSCom and the supply of medicines to 31 CSCom from December 2020. Equipment support began in March 2021 in 31 CSCom.

### Intervention logic model

**Figure 2:**
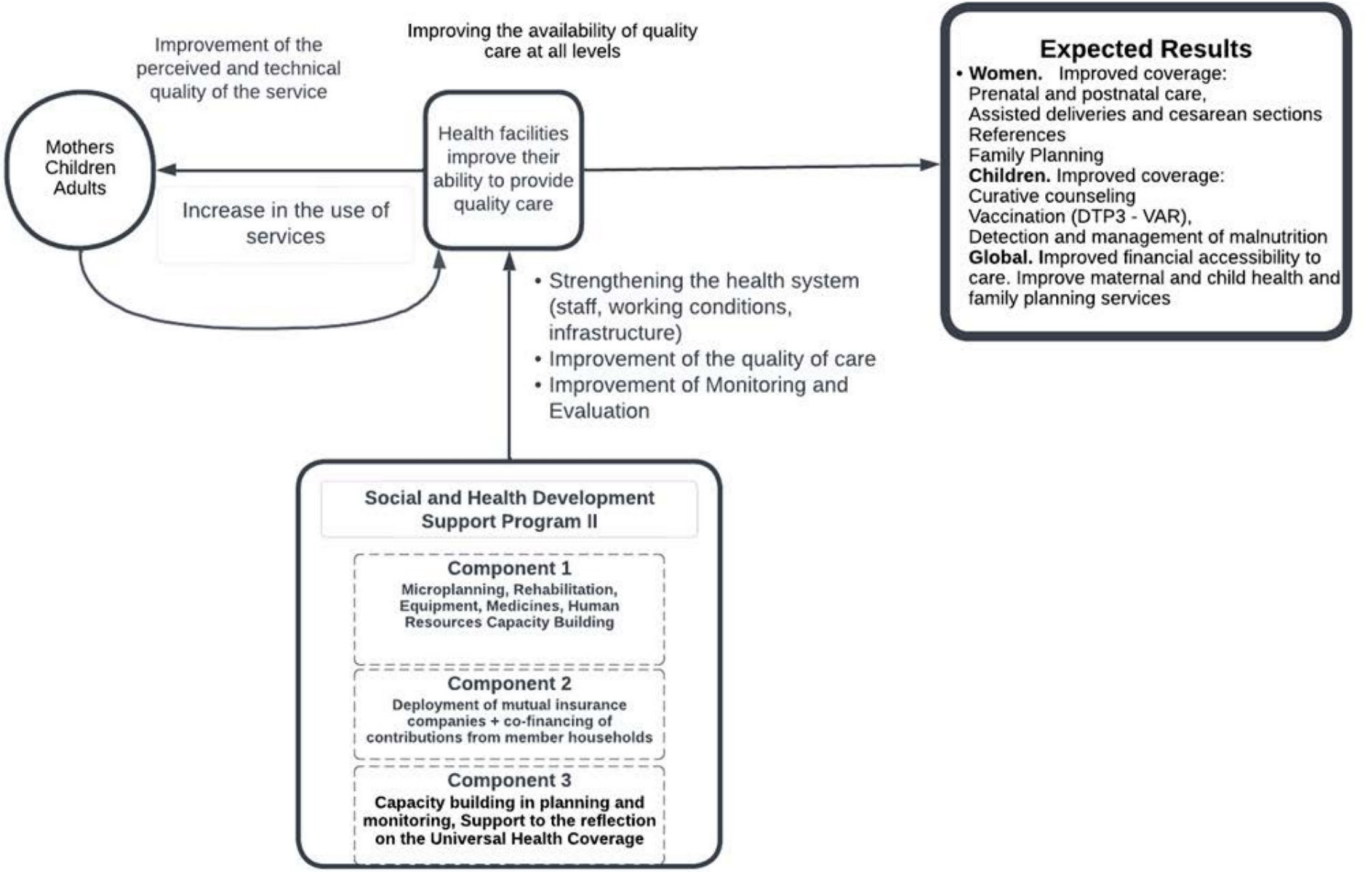
PADSS2 II intervention logic model. PADSS2, Health and Social Development Program of the Mopti Region.

The evaluation of the effects of the project falls within the scope of a theory-based evaluation, where the design and implementation of the evaluation are guided by the theory of the programme.^26^ Figure 2 shows the theory that PADSS2 could increase healthcare use among women and children under five. The intervention theory is based on international and regional research showing that the availability of equipment and medicines leads to an increase in the use of health services,^27 28^ and that perception of poor quality of training can lead to a reduction in the use of care by pregnant women.^29^ Similarly, the establishment of CBHI schemes with a strong membership subsidy and improved supply of care should cumulatively increase the ability of CSCom to provide high-quality care and lead the community to increased use of healthcare and recommend it to others.^30^

### Study design

Considering the complexity of the intervention^31^ and its phased, sequential deployment, alongside the specific characteristics of its components and the anticipated gradual response in healthcare demand, it seems unlikely that an immediate effect on service levels would occur. This assessment aligns with the principles of public health, which recognise that complex interventions often require time to manifest observable changes in health systems and outcomes.^32^

In an epidemiological framework, our analysis focuses on the potential effects of the intervention on healthcare utilisation trends. We propose hypothesis 1 (H1), suggesting that the initial phase of the intervention (C1) will have a less pronounced effect on healthcare utilisation trends compared with the subsequent phase (C2). This hypothesis is based on the assumption of reduced elasticity in the demand for care due solely to the implementation of CBHI. Furthermore, hypothesis 2 (H2) posits that the addition of component C2 to C1 is anticipated to influence positively the trends in health service attendance. Additionally, hypothesis 3 (H3) speculates that the escalation of terrorist activities may have a relatively smaller impact on the utilisation of health services in intervention districts compared with control districts, suggesting a potential mitigating effect of the intervention on service attendance in the face of increased security challenges.

We used a controlled interrupted time series design^33^ to address the intervention’s complexity, methodological challenges inherent to its intricate nature and its specific deployment strategies. The intervention group consisted of CSComs in the cercles of Mopti (n=30) and Bandiagara (n=27). The comparison group was formed from Koro (n=25) and Djenné (n=23), two cercles within the same region that did not receive the intervention. We integrated spline regression methods to investigate the intervention’s effects on healthcare utilisation trends. This approach entailed comparing monthly trends in health indicators, both prior to and during the implementation of the two components of the PADSS2 intervention, across intervention CSComs (n=57) and comparison CSComs (n=48). The observation period spanned from January 2016 to December 2021, encompassing 6 months prior to the commencement of phase C1, 24 months during C1 implementation, and 30 months throughout the implementation of phase C2.

### Data source

The data on the utilisation of maternal and child health services were sourced from the District Health Information System. The reliability of this data has been established, with its application in numerous other studies.^34 35^ To complement this, additional primary data collection was conducted, yielding precise information on the initiation and completion dates of each programme component within each intervention CSComs. This process also gathered detailed information about the contextual aspects of the CSComs, including the number and qualifications of health workers, the target population and geographic distribution.

In terms of data concerning violent events, we used the political violence database developed by the NGOs, The Armed Conflict Location & Event Data Project (ACLED).^36^ This database documents each attack or security incident, including specific details about the dates, geographical locations and the actors involved in each event. The database, as applied in our study, encompasses 767 events recorded between 2016 and 2021 in both the intervention and comparison cercles.

### Variables and measurements

The objective of PADSS2 was to enhance the utilisation of healthcare services among children, pregnant women and women of childbearing age, with the aim of improving maternal and child health. Specifically, the programme focused on several maternal health coverage indicators, including prenatal and postnatal consultations, assisted births, caesarean sections, referrals and family planning. Additionally, external consultations for children under 5 were targeted, encompassing vaccination (DTC3—VAR), malnutrition detection and management and curative counselling for this age group.

Out of these metrics, we selected five variables for our study, prioritising them based on their accessibility and completeness. These variables are detailed in table 1.

**Table 1:** Outcome variables and measurements

Results’ variables	Measures
Proportion of pregnant women receiving prenatal consultations 1 (CPN1)	Number of pregnant women receiving prenatal consultations/total number of pregnancy expected
Proportion of pregnant women receiving prenatal consultations 4 (CPN4)	Number of pregnant women receiving prenatal consultations/total number of pregnancy expected
Proportion of assisted births	Number of assisted births/total number of expected pregnancies
Proportion of new under five curative consultations	Number of new curative consultations for children under 5 years of age/total number of children under five
Cumulative proportion of curative consultation of less than 5 years (new and old)	Number of under-five curative consultations (new and old)/total number of children under five

### Geographical variable

We geolocated violent events from the ACLED database within a Geographic Information Systems (GIS) framework. We then created a variable to quantify the number of attacks or incidents occurring monthly within a 10 km diameter area surrounding each CsCom for both intervention and control groups. This area was defined in GIS by constructing a 5 km radius buffer around each CsCom. A spatial join was used to tally the violent events within these specified areas. All geographic analyses were performed with QGIS V.3.26 software.

### Analytical approach and modelling

Our analytical strategy considers multiple factors. First, PADSS2 is a multifaceted intervention impacting both the demand and supply of care. Second, the intervention’s deployment occurred in two stages: the initial phase (C1) in June 2017 and a subsequent phase (C2) in June 2019. Given the sequential nature of these phases, we anticipate gradual effects with an inherent latency, rather than immediate impacts, a hypothesis supported by visual analysis. To account for this, we employed spline regressions. These regressions ensure continuity at the transition points—before the intervention and during its two phases—thereby allowing for a directional change in trends without abrupt or unexpected shifts in indicator levels at these critical points.

We used a mixed-effects spline negative binomial regression model that leverages the adaptability of spline functions within the negative binomial regression framework. This approach facilitates the modelling of non-linear relationships between predictors and the expected count or rate of events.

Our analysis focused on assessing the impact of two distinct intervention components. We examined the ‘double difference’ in trends between the intervention and comparison groups, from the baseline to the implementation of the first component, and similarly for the second component.

Regarding the effect modifications due to the intensification of terrorist attacks, our hypothesis posits that such attacks will impact access to care, irrespective of the intervention. Given that the intervention aims to bolster the health system, we anticipate that this strengthening should enhance its resilience. Consequently, health centres in intervention districts affected by attacks are expected to show either sustained or less reduced service attendance compared with those in comparison districts.

The frequency of terrorist attacks may vary; they can increase, remain stable or decrease, occurring either sporadically or infrequently during the study period. Therefore, we determined that assessing cumulative terrorist attack frequency is the most effective method.

We also expect the effects of these attacks to manifest over several months. Accumulating attacks at a healthcare facility (CSCom) could eventually render it an insecure zone, thereby impacting health services. To analyse this, we tested a dose–response effect of terrorist attacks, categorising exposure into four levels: 0, 1–4, 5–9 and 10–40.

We also evaluated the effect modification due to the intensification of terrorist attacks in both the comparison and intervention CSComs.

To visually identify trends, seasonality, functional forms of outcome variables, outliers and interruption points between preintervention and intervention phases, we graphically represented all variables over time. Our visual analysis indicated time-dependent changes, such as delayed improvements following initial district investments.

Considering the multilevel structure of our data, which consists of monthly healthcare use measurements nested within CSComs, we employed a mixed-effects negative binomial segmented regression method.^37^ This analysis accounted for various factors, including population size, secular trend, overdispersion and seasonality,^38^ treating each month as a distinct category with a fixed parameter for seasonality magnitude.

Given that the outcome variables conform to a negative binomial distribution, we applied a non-linear combination of regression parameters to determine the intervention’s impact on health service utilisation trends. Factors such as the escalating insecurity from terrorist attacks potentially influence the outcomes of components C1 and C2. To assess how terrorist attack intensification altered the intervention effects, we included a categorical variable representing the monthly number of attacks in each CSComs’ catchment area. We then conducted a likelihood ratio test^39^ to compare models with and without the terrorist attack factor.

All fixed effects were presented as incidence rate ratios with 95% CIs.

The basic model for the negative binomial regression with a linear trend hypothesis is defined as follows:

$$\begin{array}{rl} {Outcome}_{ir}= & \beta_{0}+\beta_{1}Group+\beta_{1}trendBaseline+\beta_{3}TrendC1+\beta_{4}trendC2+Group\\ & \times \left(\beta_{5}\times trendBaseline+\beta_{6}TrendC1+\beta_{7}trendC2\right)+\beta_{3}[1-4attacks]\\ & +\beta_{9}[5-9attacks]+\beta_{1e}[10-40attacks]+Mols_{i} \end{array}$$

To operationalise the model components, we employed the Stata *mkspline* function to generate spline variables from the overall time variable. Knots were strategically placed at 18 and 42 time points to mark the commencement of phases 1 and 2 of the intervention.

The phases of interest are defined as *trendBaseline*, *trendC1* and *trendC2*, each associated with specific time intervals within the study period. The objective is to delineate the temporal influence on outcome variables, which is hypothesised to vary across these distinct phases. Specifically, *trendBaseline* quantifies the elapsed months from the study’s commencement to the onset of phase C1, and then becomes constant until the end of the observation period. TrendC1 remains 0 until the introduction of C1, thereafter tracking the months elapsed until the initiation of C2, at which point it becomes constant until the end of the observation period. TrendC2, similarly, is 0 until C2 starts, subsequently counts the number of months after its introduction until the end of the observation period. This approach facilitates a nuanced analysis of temporal effects on outcomes, accommodating non-linear time-dependent changes.

This model allowed us to estimate the following effects related to our study objectives:

-
$\beta_{3}:$ the effect of component 1 (the double difference of trend between the intervention and comparison groups from baseline to rollout of component 1).
-
$\beta_{4}:$ the effect of component 2 (the double difference of trend between the intervention and comparison groups from baseline to rollout of component 2).
-
$\beta_{8}-\beta_{10}:$ the effect of the intensification of terrorist attack in the two groups.

All statistical analyses were conducted using Stata software, V.18. We calculated incidence ratios, CI and differences in trend CIs using the ‘nlcom’ command.

### Patient and public involvement statement

Patients were not included in this study.

## Results

**Figure 3:**
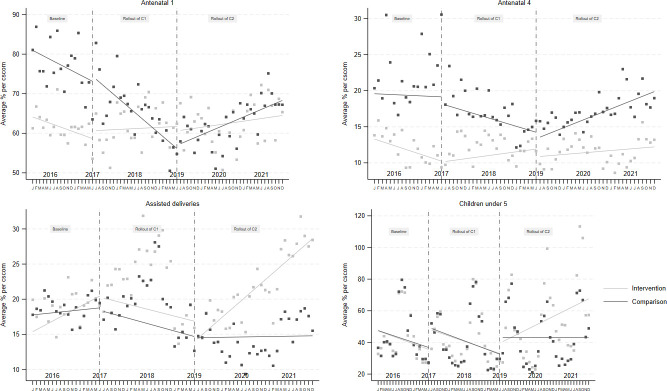
Trend in the use of maternal and child health services, created by authors.

In summary, our research applied a mixed spline regression model to thoroughly assess the PADSS programme’s impact on key maternal and child health metrics. This approach allowed us to distinctly parse out the effects attributable to the intervention itself from those influenced by terrorist activities. The main results are meticulously outlined in table 2A,B, and figure 3 underscoring the outcomes attributed to the programme.

**Table 2:** (A) Results of the negative binomial regression on the effects of PADSS2 on first and fourth antenatal visits, generated by the authors and (B) results of the negative binomial regression on the effects of PADSS2 on assisted deliveries and children under 5 visits, generated by the authors

(A)	First antenatal visits			First antenatal visits		
	Estimation	IC (95%)	p-value	Estimation	IC (95%)	p-value
Effect of PADSS						
Effect of C1	1.37	(1.28 to 1.47)	0	2.07	(1.86 to 2.28)	0
Effect of C2	−0.53	(−0.61 to −0.45)	0	−1.16	(−1.34 to −0.99)	0
Effect of terrorist attacks						
(1–4 attacks)	19.57	(−4.46 to 43.60)	0.11	4.13	(−46.26 to 54.53)	0.87
(5–9 attacks)	11.05	(−11.92 to 34.02)	0.35	−20.59	(−60.16 to 18.99)	0.31
(10–40 attacks)	1.82	(−19.59 to 23.23)	0.87	−19.59	(−60.33 to 21.15)	0.35
**(B)**	**Assisted deliveries**			**Children under 5 visits**		
	**Estimation**	**IC (95%)**	**p-value**	**Estimation**	**IC (95%)**	**p-value**
Effect of PADSS						
Effect of C1	0.39	(0.20 to 0.58)	0	0.32	(0.20 to 0.43)	0
Effect of C2	1.52	(1.36 to 1.68)	0	1.36	(1.27 to 1.46)	0
Effect of terrorist attacks						
(1–4 attacks)	−22.23	(−58.83 to 14.37)	0.23	−25.97	(−47.70 to −4.24)	0.02
(5–9 attacks)	−8.08	(−52.60 to 36.44)	0.72	−24.69	(−47.44 to −1.94)	0.03
(10–40 attacks)	−26.61	(−62.74 to ─9.52)	0.15	−39.86	(−58.33 to −21.39)	0

Specifically, the initiation of the first component of the programme corresponded with a discernible improvement in assisted deliveries, evidenced by a 0.39% increase (95% CI (0.20 to 0.58)). The implementation of the second component further augmented this trend, exhibiting a marked increase of 1.52% in the number of assisted deliveries in intervention CSComs compared with control groups (95% CI (1.36 to 1.68)). It is crucial to highlight that, in health facilities experiencing intensified terrorist activities versus those unaffected, there was no significant association between the escalation of attacks and a reduction in assisted deliveries.

### Service utilisation among pregnant women

We observed that the initiation of component 1 of the programme was associated with a significant increase in these visits: a 1.37% rise in first antenatal visits (95% CI (1.28 to 1.47)) and a 2.07% increase in fourth antenatal visits (95% CI (1.86 to 2.28)). Conversely, the implementation of component 2 appeared to inversely affect these metrics, showing a 0.53% decrease in first antenatal visits and a 1.16% decrease in fourth antenatal visits in intervention CSComs compared with controls (95% CI (−1.34 to −0.99)). Interestingly, similar to the findings on assisted deliveries, the intensification of terrorist activities did not exhibit a significant correlation with decreases in first and fourth antenatal visits, even when comparing centres with varying levels of terrorist exposure.

### Service utilisation among children

Regarding visits for children under 5, our research uncovered significant effects from both components of the programme. Component 1 led to a modest 0.32% increase in these visits (95% CI (0.20 to 0.43)), while the implementation of component 2 resulted in a more pronounced increase of 1.36% (95% CI (1.27 to 1.46)). Notably, in areas afflicted by cumulative terrorist attacks, we discerned a marked association between the intensification of these attacks and a reduction in visits for children under 5. This decline ranged from −24.69% to −39.86% in comparison to areas not impacted by terrorism.

### Discussion

In this article, we evaluated the impact of the PADSS2 programme on maternal and child health service utilisation in Mopti and Bandiagara health facilities. Additionally, we investigated how the escalation of terrorist attacks influenced the effectiveness of this intervention in healthcare usage.

Our findings indicate that the PADSS programme significantly improved outcomes in assisted deliveries, first and fourth antenatal visits and children under five visits. The notable exception was the decline in first and fourth antenatal visits observed during the implementation of component 2. Interestingly, while intensified terrorist attacks did not significantly affect assisted deliveries or antenatal visits, they were associated with a marked decrease in visits for children under 5. Although the PADSS programme positively influenced maternal and child health indicators, the overall impact was relatively modest, especially considering the programme’s comprehensive approach aimed at strengthening both the supply and demand aspects of healthcare.

This article primarily addresses the distal effects of the project, specifically focusing on the use of care by pregnant women and children, as this was the project’s declared intermediate objective.^40^ Our evaluation, guided by intervention theory,^26^ centres on analysing these specific effects on care utilisation. The relatively modest impact on care usage does not preclude the possibility of the intervention producing more significant outcomes in other areas. However, another study revealed that despite substantial medicine allocations (EUR 162 000 between 2019 and 2021),^40^ the project did not significantly impact the finances of the community health associations managing the CSComs. Further investigation is needed to confirm improvements in care quality or infrastructure.

Current evidence suggests that the project has not yet significantly influenced the intended populations. Notably, even introducing the enhanced care supply component did not markedly increase care usage, following the implementation of actions to boost care demand. To thoroughly comprehend the intervention’s limited effect, in-depth qualitative studies employing innovative methodologies are essential. An example of such an approach is the ‘process tracing’ technique used in Burkina Faso, which effectively analysed the ineffectiveness of a result-based financing intervention.^41^

Exploring the field of evaluation science regarding the limited impacts of interventions can help us identify potential explanations. These might include three types of failure: intervention failure (issues in developing the intervention content), implementation failure (problems in executing the intervention) or method failure (flaws in the techniques used to assess the intervention’s effects).^42^

In addition, the differing effects of terrorist activities on healthcare, where they seem to leave assisted deliveries and antenatal visits relatively unaffected but have a more significant impact on children under five, call for a detailed examination. This variation might be due to the varying levels of resilience in services; maternal healthcare might be more robust compared with services for children. Also, in times of crisis, communities might focus on immediate, life-saving care for expectant mothers, ensuring continued access to antenatal and delivery services, while less urgent child healthcare might be put on hold due to fear, displacement or limited resources. Moreover, terrorist activities can indirectly make healthcare less accessible for young children by disrupting transport, damaging facilities and creating an overall atmosphere of fear. It is essential to fully understand these complex interactions to create effective strategies and lessen the negative impacts of terrorism on the more vulnerable groups in the healthcare system.

## Implementation challenges

Numerous implementation challenges may have influenced the achievement of more significant results in the project. Many previous works highlighted the role of the context in implementing public health interventions in the Sahel.^22^ First, the project designers chose to act in one of Mali’s hardest-to-reach regions, where the contextual and security challenges were enormous.^24 43^ While this choice is certainly justified ethically and humanitarianly (but also programmatic since it followed another project of the same lessor Agence Française de Développement (AFD) and complemented another project on offer (Muskoka)), it made implementation very difficult. For example, the two project managers (French doctors) who succeeded each other during the project (4.5 years, the first arriving 2 years after the start of the project) were never allowed to go there and had to coordinate activities remotely (630 km). Although the region had just been liberated and accessible, the political-security context subsequently changed, making the area inaccessible to the representatives of the French donors. In addition, the political and governance context complicated the organisation of procurement, monitoring or management procedures and project steering committees (only two of the 10 planned)^40^ —often delaying things by many months. For 13 million Euros (I3S+Muskoka), the project planned for 4 years and lasted 6 years. Within this budget, the funding plan planned a division between strengthening care (5 million), improving affordability (4.5 million), capacity building (2.5 million) and miscellaneous (1 million). Finally, for the affordability component, four times less was spent on providing membership grants for populations (470 000) than on supporting CBHI deployment activities by stakeholders (USD $1.9 million).

Moreover, the project had no influence on the human resources of health centres. Yet, at the heart of the quality of care^44^ and their deficiencies were a ‘*fatal hypothesis for the project*’^40^ according to the capitalisation report. In addition to the two coups d’état during the project period (and France’s decision at the beginning of 2022 to suspend its collaboration with Mali), the COVID-19 pandemic and its impacts on the Malian health system are obviously part of the explanation,^45 46^ especially since 1.2 million Euros of the I3S project were allocated to the fight against this pandemic. While CBHI innovation has been relatively well accepted, significant challenges of coordination of the actors involved and coherence with State policies have been identified.^47^ Challenges and delays in reimbursement of healthcare providers by the CBHI organisations have been noted, thus certainly making the first reluctant to take over the beneficiaries of the project. The 2020 arrival of additional funds provided by the World Bank and its FBR project did not strengthen the financial capacity of health centres.^40^ In 2019, the state decreed the exemption from the payment of care for the two targets of the project, but it was never implemented.^3 40^ Thus, it has no influence on the results of the study.

### Strengths and methodological considerations

The originality of your paper can be distinguished on two notable fronts. Primarily, it offers a holistic evaluation of a healthcare intervention that simultaneously addresses both the demand and supply dimensions of care. This comprehensive approach sets it apart from typical evaluations within the Sub-Saharan African context, which generally focus on interventions targeting either demand or supply, but seldom both. Second, the study innovatively incorporates the increasingly pertinent issue of terrorism into its framework. In many Sahelian countries, the impact of terrorism has become an inescapable reality, necessitating its consideration in the evaluation of future public health interventions. Moreover, the study demonstrates the value of using routine data from the national health information system for complex evaluations of health programmes, as has already been the case in Mali.^9^ Our analytical approach makes an important contribution to the evaluation methodology and to the understanding of the long-term effect of system-building interventions in a Sahel context. In particular, we have shown that a description of the intervention theory and its implementation logic is an important step in identifying the appropriate impact assessment estimate and in particular the selection of an appropriate functional form of time representation, such as a time series interrupted with spline models.^48^ Despite these strengths, some limitations of the study need to be considered when interpreting the results. One of the limitations of our quasi-experimental analytical approach is that intervention and control CSComs were not randomly selected for reasons beyond the control of researchers. This lack of randomisation may have led to residual confusion and biased estimates of the effect of the intervention. While health system strengthening interventions such as PADSS2 can strengthen the resilience of health systems to shocks,^49^ we were unable to highlight the extent to which COVID-19 altered the effect of the intervention on maternal and child health indicators. However, we assumed that if there is a COVID-19 effect, it will be non-differential. In addition, we analysed the average effects for all intervention CSComs. In the absence of data on the ground and in the project capitalisation report,^40^ we could not analyse the heterogeneity of the effects of PADSS2 and the influence of the degree of the implementation of the components of the intervention in each of the CSComs on the maternal and child health outcomes targeted by the intervention. Finally, by dividing time into just three segments, we assumed that the effects of the intervention remained constant throughout the C1 and C2 segments. However, a recent publication^50^ suggests that the effects could vary over time. Unfortunately, we were unable to explore this possibility because the segments are not long enough to allow for an examination of the variability of effects within each segment.

### Relationships between donors and researchers for the evaluation of interventions

The analysis proposed in this study also shows the undeniable added value of a rapprochement between development funders and research teams. Although the study has not uncovered the major effects of intervention on the use of care, it provides insights into intervention theory and funding. Beyond the issues of conflict of interest^51^ and the cartel of success^52^ that can sometimes be seen in interventional research, the dialogue between donors and researchers remains to be built. The example of the evaluation of result-based funding in Burkina Faso, where the research estimate has adapted to local constraints and the needs of donors,^53^ in particular by means of reappearing quasi-experimental estimates,^54^ also shows the need for flexibility and adaptation of the evaluation according to the contexts, as proposed by Bamberger *et al*.^55^ It should be noted, however, that in this case, the donor (France) and the fund manager (AFD) had not planned to fund evaluative research within I3S. It was thanks to advocacy from researchers and the circumstances that made budgetary balances available that such an assessment could be carried out. However, since it was decided 4 years after the start of I3S, it was impossible to have data available to carry out an impact assessment with an experimental method or with population data. However, AFD has been promoting these methods for ages,^56^ although a recent internal discussion suggests some form of openness for other types of impact assessment methods.^57^ Not to mention their conflicts of interest, as they were involved in the intervention, consultants were recruited at the end of the project in Mali to realise a capitalisation of the project.^40^ They tried to carry out an impact assessment on healthcare indicators. However, they did not have the scientific expertise to mobilise quasi-experimental complex approaches as we tried in this study. Hopefully, this article will help to make the use of research teams able to have methods and data available to assess the impact of complex interventions more systematic.

### Conclusion

In this study, we assessed the PADSS2 programme’s effects on maternal and child health service utilisation in Mopti and Bandiagara amidst increasing terrorist attacks. Our findings reveal a significant, though modest, improvement in assisted deliveries, antenatal visits and children under five visits, but with a decline in antenatal visits during the second intervention phase. The heightened terrorist activities notably reduced visits for children under 5 but did not significantly impact other areas. The study underscores the programme’s partial success in enhancing healthcare supply and demand. However, implementation challenges, contextual complexities and diverse impacts of terrorism on healthcare services have hindered achieving more substantial results. The study suggests that future public health interventions in similar contexts should integrate rigorous implementation science and foster collaboration between development donors and research teams to enhance effectiveness.

10.1136/bmjgh-2023-012816.supp1 Supplementary data

10.1136/bmjgh-2023-012816.supp2 Supplementary data

## Data Availability

Data are available upon reasonable request. Data may be obtained from a third party and are not publicly available. Data are available upon request. The data set and statistical code are available from the corresponding author (DZ) upon reasonable request.
