# Supplementary figures and images for: Improving healthcare accessibility for pregnant women and children in the context of health system strengthening initiatives and terrorist attacks in Central Mali: a controlled interrupted time series analysis
Source: BMJ Glob Health. 2024 May 2;7(Suppl 9):e012816. doi: 10.1136/bmjgh-2023-012816 (PMC11107806; doi:10.1136/bmjgh-2023-012816)

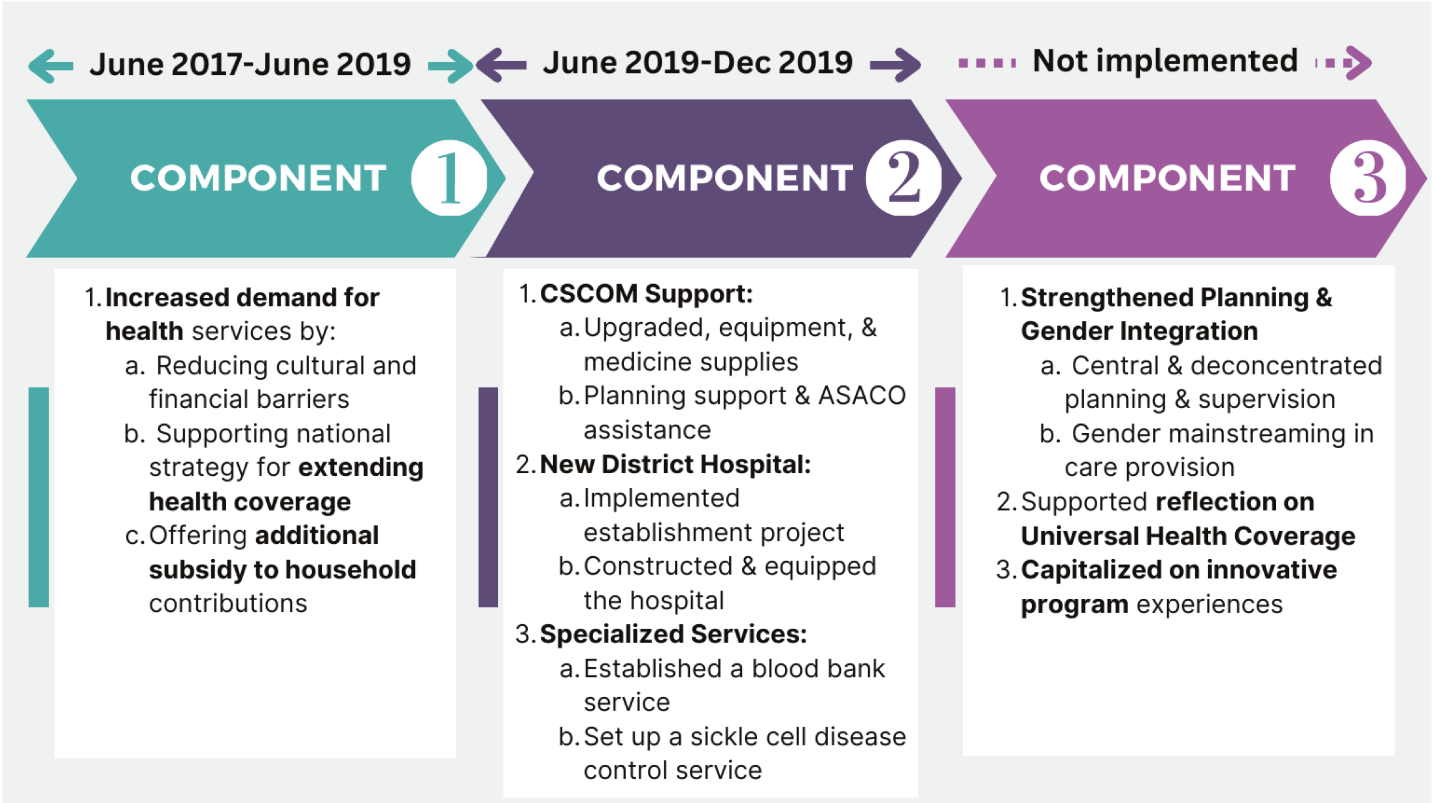

Supplement: Supplementary data [file bmjgh-2023-012816supp001.pdf]

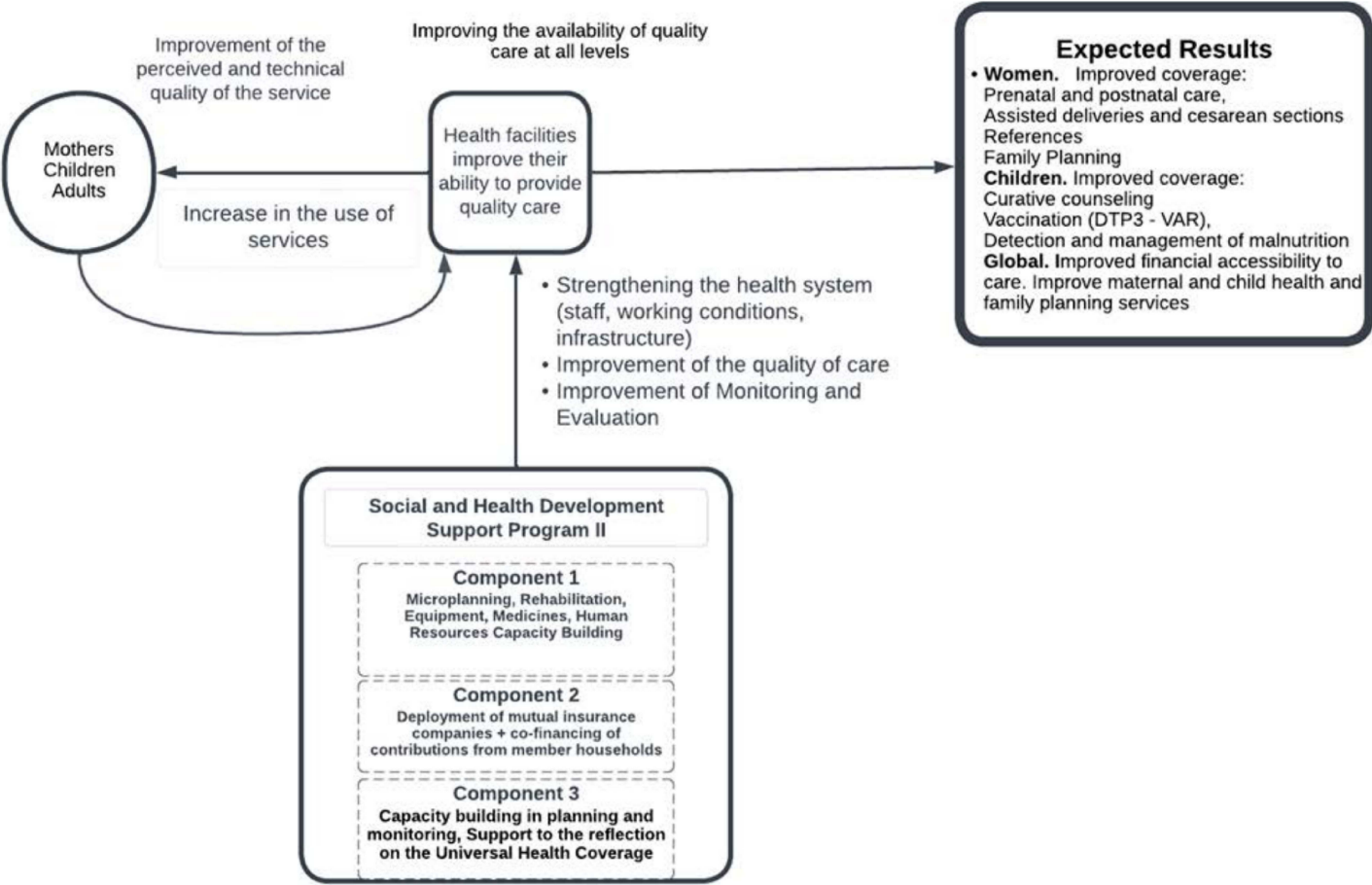

Supplement: Supplementary data [file bmjgh-2023-012816supp002.pdf]
